# Application of bioluminescence resonance energy transfer-based cell tracking approach in bone tissue engineering

**DOI:** 10.1177/2041731421995465

**Published:** 2021-02-16

**Authors:** Lufei Wang, Dong Joon Lee, Han Han, Lixing Zhao, Hiroshi Tsukamoto, Yong-IL Kim, Adele M Musicant, Kshitij Parag-Sharma, Xiangxiang Hu, Henry C Tseng, Jen-Tsan Chi, Zhengyan Wang, Antonio L Amelio, Ching-Chang Ko

**Affiliations:** 1 Division of Oral and Craniofacial Health Sciences, University of North Carolina Adams School of Dentistry, Chapel Hill, NC, USA; 2 State Key Laboratory of Oral Diseases, National Clinical Research Center for Oral Diseases, Department of Orthodontics, West China Hospital of Stomatology, Sichuan University, Chengdu, China; 3 Research & Development Center, Nitta Gelatin Inc., Yao-City, Osaka, Japan; 4 Department of Orthodontics, School of Dentistry, Pusan National University, Yangsan, South Korea; 5 Graduate Curriculum in Genetics and Molecular Biology, University of North Carolina School of Medicine, Chapel Hill, NC, USA; 6 Graduate Curriculum in Cell Biology and Physiology, University of North Carolina School of Medicine, Chapel Hill, NC, USA; 7 Duke Eye Center and Department of Ophthalmology, Duke University Medical Center, Durham, NC, USA; 8 Department of Molecular Genetics and Microbiology, Center for Genomics and Computational Biology, Duke University Medical Center, Durham, NC, USA; 9 Department of Pediatric Dentistry, University of North Carolina Adams School of Dentistry, Chapel Hill, NC, USA; 10 Department of Cell Biology and Physiology, University of North Carolina School of Medicine, Chapel Hill, NC, USA; 11 Division of Orthodontics, The Ohio State University College of Dentistry, Columbus, OH, USA

**Keywords:** Bioluminescent imaging, cell tracking, bone tissue engineering, bioluminescence resonance energy transfer, mesenchymal stem cells

## Abstract

Bioluminescent imaging (BLI) has emerged as a popular in vivo tracking modality in bone regeneration studies stemming from its clear advantages: non-invasive, real-time, and inexpensive. We recently adopted bioluminescence resonance energy transfer (BRET) principle to improve BLI cell tracking and generated the brightest bioluminescent signal known to date, which thus enables more sensitive real-time cell tracking at deep tissue level. In the present study, we brought BRET-based cell tracking strategy into the field of bone tissue engineering for the first time. We labeled rat mesenchymal stem cells (rMSCs) with our in-house BRET-based GpNLuc reporter and evaluated the cell tracking efficacy both in vitro and in vivo. In scaffold-free spheroid 3D culture system, using BRET-based GpNLuc labeling resulted in significantly better correlation to cell numbers than a fluorescence based approach. In scaffold-based 3D culture system, GpNLuc-rMSCs displayed robust bioluminescence signals with minimal background noise. Furthermore, a tight correlation between BLI signal and cell number highlighted the robust reliability of using BRET-based BLI. In calvarial critical sized defect model, robust signal and the consistency in cell survival evaluation collectively supported BRET-based GpNLuc labeling as a reliable approach for non-invasively tracking MSC. In summary, BRET-based GpNLuc labeling is a robust, reliable, and inexpensive real-time cell tracking method, which offers a promising direction for the technological innovation of BLI and even non-invasive tracking systems, in the field of bone tissue engineering.

## Introduction

Reconstruction of critical-sized bone defects remains a challenging procedure for orthopedic surgeon and causes a staggering financial burden on the healthcare system. Stem cell-based bone tissue engineering has been suggested as a promising approach for reconstructing bone defects and may serve as an alternative to bone graft.^1^ The emergence of bio-compatible 3D printing techniques allow high throughput fabrication of 3D scaffolds and complex tissue constructs to be used in customized bone tissue engineering and precision medicine.^2^ Mesenchymal stem cells (MSCs) are widely utilized in bone defect repair research due to their easy isolation procedure and established regenerative potential.^3^ How to conveniently track these bioengineered stem cells following implantation and monitoring their dynamic regenerative capacities remains a key hurdle towards their application in bone defect repair. To date, many non-invasive imaging methods for spatiotemporally tracking implanted cells in vivo have emerged, for example, fluorescent or luminescent labeling, radioactive labeling (for single-photon emission computed tomography (SPECT) or positron emission computed tomography (PET)), and paramagnetic labeling (for magnetic resonance imaging (MRI)).^4,5^ Chief among these imaging modalities, bioluminescence imaging (BLI) has garnered much attention in recent years because it does not need cell-exogenous light excitation and thus eliminates critical caveats associated with tissue penetrance, autofluorescence and photo-bleaching that plague fluorescence imaging approches.^6–8^ By virtue of these properties, BLI offers robust sensitivity, deeper imaging depth, and higher signal-to-background ratio compared to fluorescence imaging. In addition, cost and time saving benefits compared to SPECT, PET and MRI approaches popularize the application of BLI in in vivo cell tracking.^9,10^

In the field of bone tissue engineering, BLI is popular for longitudinally tracking implanted tissue-engineered constructs in vivo. However, bone tissues are usually deep in the body and composed of a large portion of minerals, which weakens bioluminescent signal intensity and compromises the resolution by tissue scattering and absorption.^11^ Therefore, BLI would sometimes need to associate with another reporter gene, like enhanced green fluorescent protein (eGFP), either to enhance the signal or to double-confirm its specificity by another imaging approach.^12–15^ If using 3D printed scaffold, much less BLI study has been seen probably because mineral deposition resulted from osteogenic differentiation within and around the 3D porous scaffolds may further hinder bioluminescent signal.

To improve above situations, a chimeric eGFP-NanoLuc (GpNLuc) fusion reporter protein coined LumiFluor, which has been created by us and previously displayed great potential in in vivo monitoring tumorigenesis, seems like a promising candidate.^16^ First, GpNLuc reporter is fluorescence-bioluminescence bifunctional, which combines the benefits of eGFP with the extremely bright (>150-fold brighter than firefly or *Renilla* luciferase), stable bioluminescent light generated by an enhanced small luciferase subunit (NanoLuc) of the deep-sea shrimp *Oplophorus gracilirostris*.^17^ Second, GpNLuc reporter is designed as bioluminescence resonance energy transfer (BRET)-based (BRET ratio: 2.60 ± 0.02), which enables the intramolecular energy transfer between NanoLuc and eGFP to generate the brightest bioluminescent signal known to date, with enhanced intensity, sensitivity, and deep tissue penetration capabilities.^16^ Third, unlike commonly used firefly luciferase which is adenosine triphosphate (ATP)-dependent, GpNLuc reporter component NanoLuc is ATP-independent. Since bone healing is very energy consuming and the ATP level has been reported to correlate with the degree of bone repair,^18^ being ATP-independent may make GpNLuc reporter a more objective and accurate tool in bone regeneration studies. Thus, following the success in in vivo cancer cell tracing, we herein utilized this novel BRET-based GpNLuc reporter to label MSCs and tested the efficacy of MSC tracking during osteogenic differentiation in vitro and in vivo (rat calvarial critical sized defect (CSD) model), which offered an improved, robust, inexpensive bioluminescent cell tracking strategy for bone tissue engineering.

## Materials and methods

### rMSC isolation, transduction with GpNLuc, flow cytometry characterization

**Figure 1.**
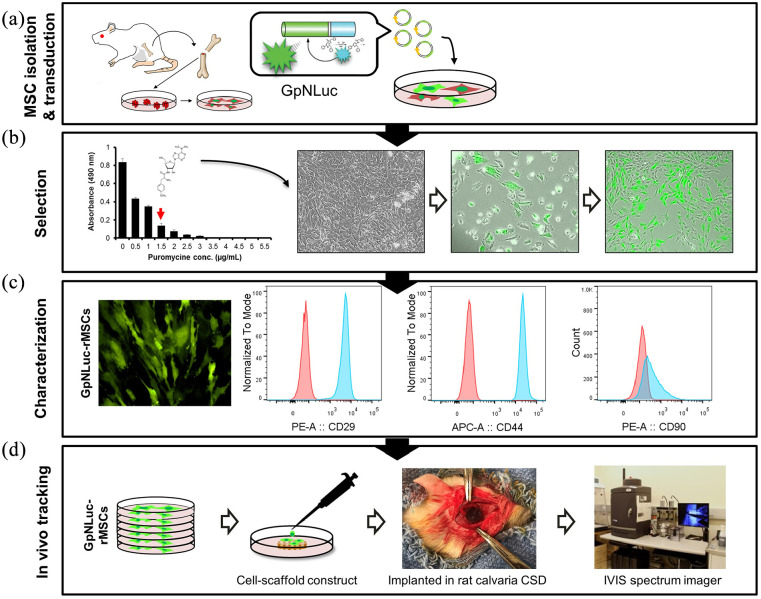
Schematic diagram of the study and establishment of GpNLuc-rMSCs: (a) MSCs were isolated from bone marrow, transduced with GpNLuc, (b) 1.5 µg/mL puromycin was used to select transduced cells according to the kill curve result. Successful expression of GpNLuc was determined by the presence of green fluorescence, (c) GpNLuc-rMSC fluorescent image and flow cytometry detection of surface markers. Blue peak: GpNLuc-rMSC sample. Red peak: isotype control, and (d) validated GpNLuc-rMSCs were used to generate cell-scaffold constructs, implanted in a rat calvaria CSD model, and monitored using In Vivo Imaging System (IVIS) spectrum imager.

Animal studies were carried out according to a protocol approved by the Institutional Animal Care and Use Committee at the University of North Carolina at Chapel Hill (protocol No. 15-273). As shown in schematic diagram Figure 1, Sprauge-Dawley rats (Charles River, USA; about 300 g, 12 weeks old) were euthanized for isolating MSCs. The femurs were removed, both ends of the femur were cut, and then bone marrow was flushed out with complete growth medium (DMEM (Invitrogen, USA) supplemented with 10% fetal bovine serum, 1% penicillin and streptomycin, and 1% Glutamax). After 24 h, non-adherent cells were removed and adherent cells were maintained in culture. Growth media was changed every 3 days. The rMSCs were plated onto 12-well tissue culture plates at a density of 1.5 × 10^5^ cells per well. After 24 h, the rMSCs were transduced using packaged lentivirus containing DNA casettes for constitutively expression of GpNLuc (Addgene #135935) and Puromycin^R^ resistance, as described elsewhere.^16,19^ Mock transfection was also performed. The “rMSCs” control when compared to the “GpNLuc-rMSCs” group throughout this study refers to mock-transfected rMSCs. Growth media was replaced 24 h post-transduction. The cells were selected with puromycin (1.5 μg/mL) for 6 days and then expanded in 100 mm tissue culture dishes. Successful expression of GpNLuc was determined by the presence of green fluorescence. For MSC surface markers verification, GpNLuc-rMSCs were characterized for CD29, CD44 and CD90 as well as CD34 and CD45 using BD Accuri™ C6 plus flow cytometer (BD Life Sciences, USA). Corresponding fluorophore conjugated antibodies and isotype controls were used for flow cytometry. Data was analyzed by FlowJo v10 software (BD Life Sciences, USA).

### MTS cell viability assay

The viablility of the rMSCs and GpNLuc-rMSCs in growth or osteogenic medium was assessed using tetrazolium dye MTS cell viability assay kit (Promega, USA) following the company’s instructions. At indicated time points, cells were incubated with MTS solution at 37°C for 1 h. The absorbance at 490 nm was measured using the Cytation 5 imaging reader (BioTek, USA).

### In vitro osteogenic differentiation assessment (RT-qPCR, ALP activity, Alizarin Red staining)

For osteogenic induction, rMSCs and GpNLuc-rMSCs were cultured in osteogenic medium (complete growth medium supplemented with 10 mM *β*-glycerolphosphate (Sigma-Aldrich, USA), 0.2 mM ascorbic acid (Sigma-Aldrich, USA), and 0.1 M dexamethasone (Sigma-Aldrich, USA)) for up to 28 days. Cells were harvested for below assays at indicated time points.

For osteogenic genes detection, RNA was extracted using Trizol (Invitrogen, USA) and reverse transcription was performed using iScript cDNA synthesis kit (Bio-Rad, USA). RT-qPCR was performed on StepOnePlus Real-time PCR system (Applied Biosystems, USA) using the iTaq™ Universal SYBR Green Supermix reagent (Bio-Rad, USA). The relative expression of target genes (*Bsp* and *Opn*) was normalized to *β-actin* based on the ΔΔCT method. The primers are listed in Supplemental Table S1.

For ALP activity detection, cells were lysed and assayed using a commercial ALP assay kit (Abcam, UK), followed by immediate optical density (OD) measurement at 405 nm using the Cytation 5 imaging reader (BioTek, USA).

For mineralization assessment, the cells were fixed with 70% cold ethanol for 30 min and stained with 1% Alizarin Red solution (Sigma-Aldrich, USA) for 10 min. After capturing images, we extracted the stained plates with 1 mL of 1% (w/v) cetylpyridinium chloride solution (Sigma-Aldrich, USA) for 10 min, and measured the OD at 562 nm as quantification outcome.

### 3D cell spheroid formation (forced aggregation)

3000 rMSCs or GpNLuc-rMSCs were seeded on 96-well round-bottom ultra-low attachment plates. A forced aggregation method was used to prepare 3D cell spheroids.^20,21^ Briefly, cell suspension was centrifuged at 1400 rpm for 4 min to allow the cells to form aggregates over time. After growing in complete growth medium for 3 days, aggregates were transferred to 24-well ultra-low attachment plates (10 aggregates per well) and switched to osteogenic medium. Osteogenic medium were refreshed every 3 days for continual osteogenic induction. Aggregates were imaged using a Nikon Eclipse Ti-U inverted microscope with bright field and fluorescence mode. The diameter and area of the aggregates was quantified using ImageJ software (NIH, USA). Fluorescence intensity (RFU) was measured using the Cytation 5 imaging reader (BioTek, USA).

### In vitro and in vivo BLI

Real-time BLI was performed using an In Vivo Imaging System (IVIS) Spectrum imager (PerkinElmer, USA) following the administration of furimazine—Nano-Glo Luciferase Assay substrate (Promega #N1120). For all in vitro BLI, cells were treated with 50 µM furimazine in Nano-Glo Luciferase Assay Buffer (Promega #N1120) for 5 min according to the manufacturer’s protocol. Then Bioluminescent images were captured with an open filter, binning set to 4. For in vivo BLI, animals were anesthetized with isoflurane prior to the subcutaneous injection of 250 µM furimazine (Promega #N1120; 1/20 dilution) into the calvaria defect site. Images were captured with an open filter, binning set to 4, and acquisition times of 60 s at the indicated settings. All BLI signal detected (both in vitro and in vivo) using the GpNLuc reporter represent BRET signal deriving from intramolecular energy transfer between NanoLuc and eGFP. Total flux (p/s) and average radiance (p/s/cm^2^/sr) were calculated using the Living Image software (PerkinElmer, USA).

### Cell-scaffold constructs generation

3D mold printing technique was utilized to fabricate PDHC scaffold as previously described.^22,23^ After sterilization, PDHC scaffold and Gelfoam^®^ (Pfizer, USA) were pre-wetted in culture medium overnight. Indicated numbers of rMSCs or GpNLuc-rMSCs were suspended in 20 µL Matrigel^®^ Matrix (Corning, USA) and seeded on PDHC scaffold and Gelfoam^®^. The cell-scaffold constructs were incubated for 15 min to allow gel attachment and infiltration, and then cultured in osteogenic medium for 28 days. Medium was refreshed every 3 days.

### Scanning electron microscopic (SEM) analysis

The rMSC and GpNLuc-rMSC-seeded PDHC scaffolds were fixed in a 2.5% glutaraldehyde/0.1 M sodium cacodylate solution (pH 7.4) for 6 h at room temperature. After critical point drying by dehydrating in an ethanol-graded series, samples were sputter-coated and imaged using a Hitachi S-4700 cold cathode field emission SEM (Hitachi High Technologies America, Inc., USA).

### Calvarial CSD model

For in vivo implantation, 1 × 10^6^ differentiated cells were suspended in Matrigel^®^ Matrix (Corning, USA) and seeded on PDHC or Gelfoam to generate cell-scaffold constructs as described earlier in this study. Four groups of construct were prepared: rMSCs-PDHC, GpNLuc-rMSCs-PDHC, rMSCs-Gelfoam, and GpNLuc-rMSCs-Gelfoam. A well-defined calvarial CSD model was performed on a total of 12 (*n* = 3) male Sprague-Dawley rats (Charles River, USA; about 300 g, 12 weeks old). The surgical procedure has been well described previously.^24^ Briefly, rats were anesthetized with an intraperitoneal injection of Ketamine/Xylazine (Puteney Inc., USA). In the center of the calvaria, an 8 mm diameter defect was created using a low-speed trephine burr and replaced by a cell-scaffold construct. Pain management and infection control medications were performed post-surgery. The rats were ad lib-fed and euthanized 8 weeks post-surgery.

### Histological analyses

After animal euthanization, calvaria was collected for histological analyses. For mineral formation evaluation, undecalcified sections were prepared.^22,24^ Explanted samples were dehydrated and infiltrated with resin (Technovit, Germany). The samples were embedded with light curable polymer embedding kit (Technovit, Germany), sectioned using EXAKT model 310 pathology saw (EXAKT Advanced Technologies, Germany) and ground to ~70 µm thickness using EXAKT Model 400 microgrinding system. Completed slides were stained using Van Gieson’s stain. Images were acquired with a Nikon Eclipse Ti-U inverted microscope and the percentage of NFB was analyzed using ImageJ software (NIH, USA).

For eGFP immunohistochemical staining, samples were fixed, decalcified, embedded, and cryosectioned to 7 μm. The slides were blocked and then incubated with anti-eGFP primary antibody (Abcam, UK) and HRP-conjugated secondary antibody. After DAB reaction (Abcam, UK), images were acquired using a Nikon Eclipse Ti-U inverted microscope.

### Statistical analysis

Experiments were performed in triplicate and repeated three times. Data was presented as mean ± standard error of the mean (SEM). Statistical analyses were performed using GraphPad Prism 5 (GraphPad Software, USA). Student’s *t* test was used for single comparisons; one-way ANOVA was used for multiple comparisons; linear and nonlinear regression was performed to test the strength of the relationship between two variables. *p* < 0.05 was set for significance.

## Results

### GpNLuc expressing rMSCs exhibit normal proliferative potential and osteogenic capacity

**Figure 2.**
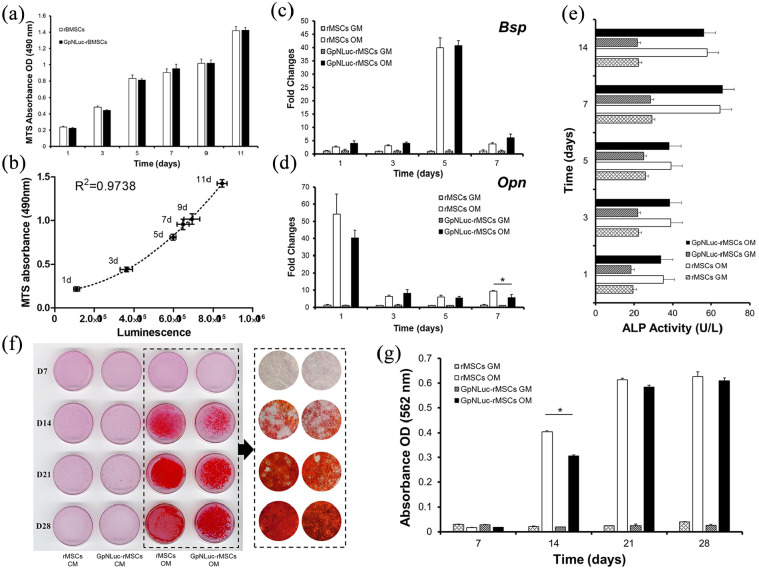
Characterization of GpNLuc-rMSCs: (a) cell viability was detected using MTS method at various time points, (b) GpNLuc-rMSCs luminescence intensity was measured after treating with 50 µM furimazine in Nano-Glo Luciferase Assay Buffer for 5 min at each time point. Polynomial regression was performed to explain the relationship between the luminescence intensity and the MTS absorbance. For (c–g), osteogenic medium was used for osteogenic induction, (c and d) Relative genes expression was normalized to *β-actin*, (e) ALP activity assay, and (f and g) mineral nodule formation was visualized by Alizarin Red staining. Absorbance was measured after CPC extraction. GM: growth medium; OM: osteogenic medium. All experiments *n* = 5. Data shown as mean ± SEM. *p* < 0.05.

Since GpNLuc reporter actually contains an eGFP expression cassette, successful GpNLuc expression can easily be determined by the presence of green fluorescence. After isolation, transduction, and selection (Figure 1(a) and (b)), established GpNLuc-rMSCs displayed normal MSC morphology and constitutively expressed GpNLuc (Figure 1(b) and (c)). About MSC surface markers verification (Figure 1(c)), GpNLuc-rMSCs expressed CD29 (99.9% ± 0.06%), CD44 (99.9% ± 0.09%), and CD90 (60.6% ± 5.1%) and did not express CD34 or CD45 (data not shown). No significant difference in cell viablility was found between rMSCs and GpNLuc-rMSCs, indicating GpNLuc-rMSCs viability was not altered by GpNLuc reporter expression (Figure 2(a)). GpNLuc-rMSCs bioluminescence capability works well: a well-fitting regression (*R*^2^ = 0.9738) was shown between the bioluminescence intensity and the number of cells (reflected by MTS absorbance) (Figure 2(b)). We next checked the osteogenic capability of GpNLuc-rMSCs by RT-qPCR, alkaline phosphatase (ALP) activity assay, and Alizarin red staining. rMSCs and GpNLuc-rMSCs were cultured in osteogenic medium (OM) for osteogenic induction. There is generally no difference in the osteogenic genes expression (*Bsp* and *Opn*) between rMSCs and GpNLuc-rMSCs at various time points, except for *Opn* expression on day 7 (Figure 2(c) and (d)). Similarly, ALP activity of GpNLuc-rMSCs was also not changed comparing to rMSCs (Figure 2(e)). Mineral nodule formation was detected using Alizarin red staining followed by absorbance quantitative data using CPC extraction (Figure 2(f) and (g)). In general, there is no significant difference in mineralization capacity between rMSCs and GpNLuc-rMSCs, except on day 14. Taken together, GpNLuc-rMSCs displayed the same behaviors as control rMSCs, indicating that GpNLuc-rMSCs are “healthy” and “normal” in term of MSC.

### BRET-based BLI offers enhanced accuracy at tracking rMSC in scaffold-free spheroid model

**Figure 3.**
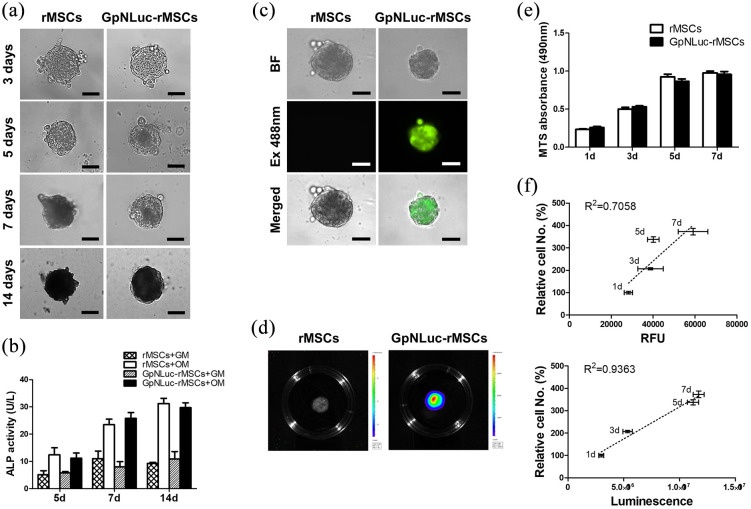
GpNLuc-rMSCs in vitro imaging in spheroid model. rMSCs and GpNLuc-rMSCs spheroids received osteogenic medium for osteogenic induction: (a) bright field imaging for morphological evaluation. Scale bar is 100 µm, (b) ALP activity assay. GM: growth medium. OM: osteogenic medium, (c) fluorescent imaging and (d) BLI for monitoring GpNLuc-rMSCs spheroids. Scale bar is 100 µm, (e) MTS cell viability assay, and (f) linear regression for analyzing the relationship between relative cell number and RFU or luminescence intensity. 1 day, 3 days, 5 days, 7 days data were entered into the regression model. Relative cell number was calculated based on the data in (e). All experiments *n* = 6. Data shown as mean ± SEM.

After the successful establishment and characterization of GpNLuc-rMSCs, we first evaluated its BLI efficacy in a scaffold-free 3D culture system—MSC spheroids, which is formed using a forced aggregation method.^17,18^ It has been demonstrated that MSC spheroid model can effectively maintain and enhance MSC multipotent differentiation potential.^25^ After aggregated, rMSCs and GpNLuc-rMSCs spheroids were cultured in osteogenic medium for osteogenic differentiation. Across various time points the morphology and size of rMSCs and GpNLuc-rMSCs spheroids generally looks the same (Figure 3(a)), which is further demonstrated by diameter and area measurement (Supplemental Figure S1). Osteogenic activity, reflected by ALP activity, also showed no significant difference between rMSCs+OM and GpNLuc-rMSCs+OM (Figure 3(b)). Lastly, GpNLuc-rMSC spheroid progression was longitudinally tracked via both fluorescence (Figure 3(c)) and BLI (Figure 3(d)) during osteogenic differentiation. At different time points during osteogenic differentiation (1–7 days), we measured MTS absorbance, fluorescence, and bioluminescence intensity of GpNLuc-rMSCs. We performed linear regression (Figure 3(f)) to examine the strength of relationship between relative cell number (calculated based on MTS absorbance in Figure 3(e)) and relative fluorescence units (RFU) or bioluminescence intensity. BLI displayed a much better linear relationship (*R*^2^ = 0.9363) with relative cell number comparing to RFU (*R*^2^ = 0.7058), suggesting that BRET-based approach has better accuracy than fluorescence approach in monitoring rMSCs in scaffold-free spheroid 3D culture system.

### BRET-based BLI enables long term tracking of rMSC in scaffold-based 3D culture systems

**Figure 4.**
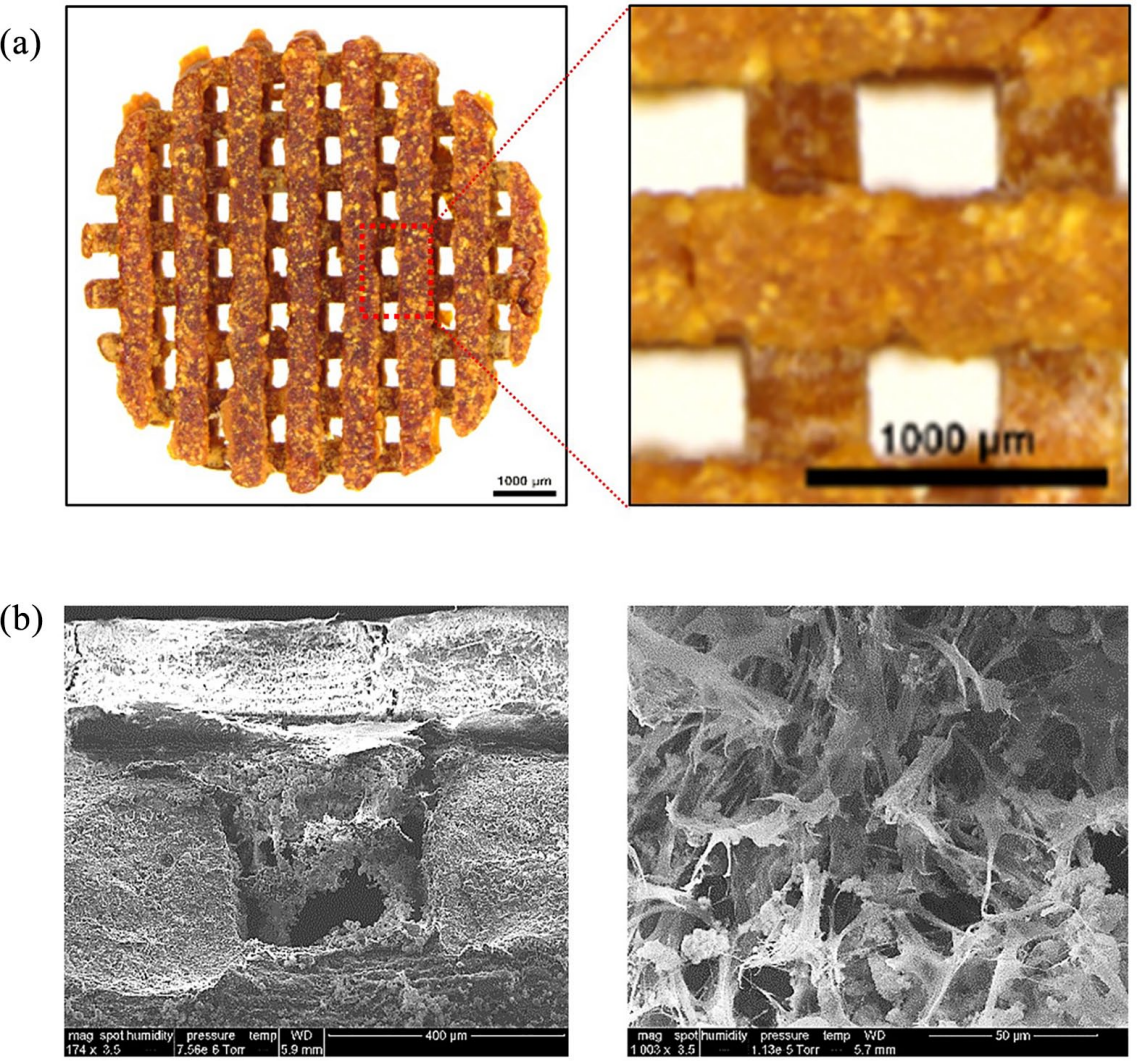
PDHC scaffold architecture and cell attachment: (a) PDHC scaffold architecture and (b) SEM imaging of GpNLuc-rMSCs infiltration and attachment.

**Figure 5.**
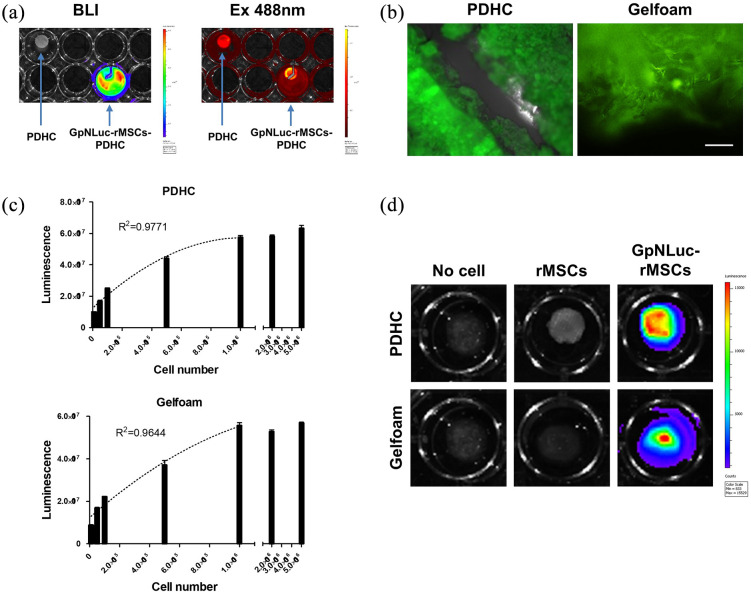
GpNLuc-rMSCs in vitro monitoring in PDHC and Gelfoam scaffold: (a) BLI and fluorescence imaging (excitation light 488 nm) of PDHC alone and GpNLuc-rMSCs seeded PDHC, (b) autofluorescence of PDHC and Gelfoam deterred the visualization of GpNLuc-rMSCs, (c) polynomial regression for evaluating the relationship between luminescence intensity and seeded cell number in PDHC or Gelfoam. *n* = 3, and (d) BLI imaging of GpNLuc-rMSCs seeded PDHC and Gelfoam after 28 days of osteogenic differentiation. Data shown as mean ± SEM.

Modern 3D printing techniques have facilitated the fabrication of nano-scaffolds for tissue engineering by enabling precise control of scaffold architecture, such as pore size, dimensions, and shape. We recently developed a 3D printed polydopmaine-laced hydroxyapatite collagen (PDHC) scaffold, which exhibited great osteoconductive potential both in vitro and in a calvaria CSD model.^22–24^ Our PDHC scaffold displayed porous architecture, which is beneficial for cell infiltration, mineral deposition, and capillary ingrowth (Figure 4(a)). Next, we seeded GpNLuc-rMSCs on the PDHC scaffold to generate cell-scaffold constructs in an attempt to evaluate the in vitro BLI efficacy. GpNLuc-rMSCs established successful attachment to the PDHC scaffold, which was confirmed by SEM (Figure 4(b)). After infiltrating into the PDHC scaffold, GpNLuc-rMSCs were still able to generate robust bioluminescence signal (Figure 5(a)). PDHC scaffold alone did not have background signal when measuring bioluminescence, however it absorbed light at 488 nm and generated autofluorescence (Figure 5(a)). The auto-fluorescence of the PDHC scaffold deterred the visualization of seeded GpNLuc-rMSCs using fluorescence imaging (Figure 5(b)). We added a transparent Gelfoam control group but it seemed that transparent Gelfoam also has the autofluorescence issue (Figure 5(b)), indicating it is better to use BLI instead of fluorescence for cell tracking in cell-scaffold constructs to minimize interfering signals.

We seeded a variety of numbers of cells on the PDHC or Gelfoam scaffold, to test the correlation between cell number and luminescence intensity. A well fitting regression was found between seeded cell number and luminescence intensity in PDHC (*R*^2^ = 0.9771) or Gelfoam (*R*^2^ = 0.9644) group. Moreover, the bioluminescence signal can be robustly detected after 28 days of osteogenic differentiation (Figure 5(d)). Taken together, robustly detectable signal, no background noise, and well correlation with cell number highlighted the superiority and reliability of using BRET-based GpNLuc reporter for rMSCs tracking in scaffold-based 3D culture systems.

### GpNLuc-rMSCs in vivo monitoring in calvaria CSD

**Figure 6.**
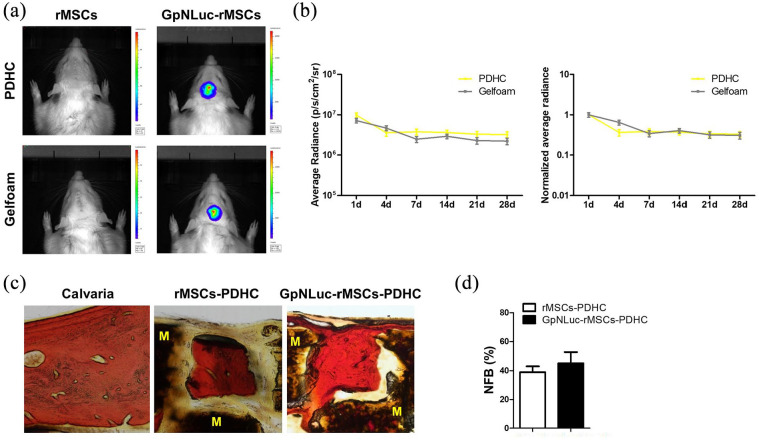
GpNLuc-rMSCs in vivo monitoring in rat calvaria CSD: (a) BLI for monitoring the implanted cells at the defect site. Twenty-eight days images were shown as representative, (b) bioluminescent intensity over time (1–28 days). The left panel showed average radiance; the right panel was plotted after normalized to those of 1 day, and (c and d) Van Gieson staining for detecting NFB within PDHC scaffold. M: material (PDHC). All experiments *n* = 3. Data shown as mean ± SEM.

Rodents are considered one of the first-choice models for in vivo bone regeneration studies because they are easy to handle and cost saving.^26^ The rat calvaria CSD model is well defined for investigating bone tissue engineering and has been tested for non-invasive bioluminescence imaging.^27,28^ An 8 mm diameter defect was created in the center of the calvaria according to the protocol described previously.^24^ After the implantation of cell-scaffold constructs, our animals were overall healthy (Supplemental Figure S2), with no systemic disease observed throughout the entire experiment period. BLI was performed to longitudinally monitor the survival of the implanted rMSCs at the defect site. Robust bioluminescence signals can be detected over extended periods of time (images on 28 days were shown as representative in Figure 6(a)). Reflected by the bioluminescence signal intensity shown in Figure 6(b), in both PDHC and Gelfoam groups, implanted cells exhibited a similar survival pattern characterized by an initial decrease followed by long-term stabilization, which is consistent with Dégano et al.^12^ study. The cell death occurred during the initial stage may result from post-injury inflammation and blood vessels lost that reduces nutrient supply. The difference in average radiance between PDHC and Gelfoam groups may be due to differential optical or penetration properties of materials (Figure 6(b), left panel). Thus, we performed normalization analysis relative to those of day 1 and found that the curves of PDHC and Gelfoam match well especially in latter time points (Figure 6(b), right panel), suggesting the consistency of BRET-based GpNLuc in cell survival evaluation across differential scaffold materials.

**Figure 7.**
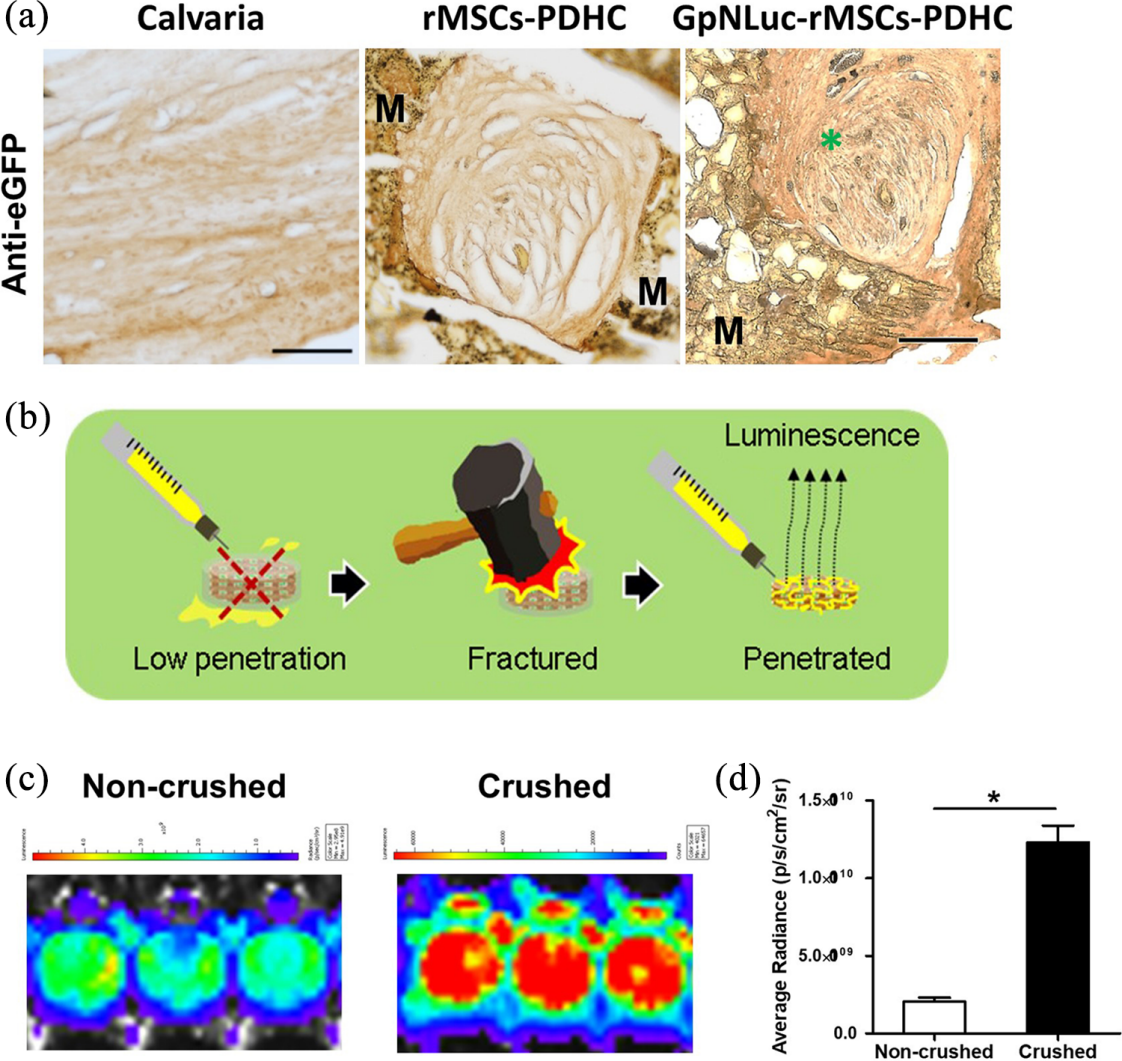
GpNLuc-rMSCs survival and colonization: (a) anti-eGFP immunohistochemical staining. M: material (PDHC). Green asterisk indicates GFP positive, (b) schematic drawing: crush the scaffold for better substrate penetration and luminescence signal, and (c and d) BLI measurement of non-crushed and crushed GpNLuc-rMSCs-PDHC explants. All experiments *n* = 3. Data shown as mean ± SEM. *p < 0.05.

Eight weeks post-surgery, the newly formed bone (NFB) within the scaffold were visualized by Van Gieson staining. GpNLuc-rMSCs group showed considerable new bone filling within the pore of PDHC and osseointegration at the NFB-material interface (Figure 6(c)). GpNLuc-rMSCs exhibited the same new bone formation capability as control rMSCs (Figure 6(d)). Anti-eGFP immunohistochemical staining revealed the survival and extensive colonization of GpNLuc-rMSCs in the NFB (Figure 7(a)). To further confirm the survival of GpNLuc-rMSCs, we also extracted GpNLuc-rMSCs-PDHC constructs from the calvaria for explant culture, and measured the bioluminescent intensity after furimazine incubation (Figure 7(b)–(d)). Since the NFB within the scaffold may obstruct furimazine penetration, we also tried crushing the scaffold, incubated with furimazine, and measured bioluminescent intensity again. Surprisingly, the bioluminescent signal from crushed samples was much stronger than that from non-crushed samples, implying that effective substrate delivery may improve in vivo BLI performance in bone tissue engineering. Taken together, our data suggested that BRET-based GpNLuc labeling is a reliable and convenient method for in vivo non-invasive MSC tracking in calvaria CSD.

## Discussion

To the best of our knowledge, the present study introduced BRET-based cell tracking strategy to the field of bone tissue engineering for the first time. We labeled rMSCs with a BRET-based GpNLuc reporter and tested the cell tracking efficacy both in vitro and in vivo. A number of advantages were found based on the experiment results, highlighting BRET-based GpNLuc labeling as a revolutionary cell tracking strategy in the field of bone tissue engineering. First, in scaffold-free spheroid 3D culture system, using BRET-based GpNLuc reporter resulted in a better correlation to cell number than fluorescence approach, which indicates a better accuracy. Second, in scaffold-based 3D culture system, BRET-based GpNLuc labeling generated robustly detectable signal and avoided the issue of background noise, suggesting its broad applicability and robust reliability. Third, in calvaria CSD model, the robust signal and the consistency in evaluating cell survival collectively supported BRET-based GpNLuc labeling as a reliable in vivo non-invasive MSC tracking method.

Recent years have seen a surge in the application of BLI in the tissue engineering for bone wound healing and bone defect repair.^10,11^ Given the advantages of non-invasive, real-time, and inexpensive, BLI has indeed facilitated the tracking of cells or biological processes in bone regeneration and hence promoted the advancement of bone tissue engineering. In addition, the lower cost and equipment demand make BLI more popular in pre-clinical studies, even though CT and MRI may offer higher resolution and deeper imaging depth.^10^ It has been demonstrated that biological sources of light has sufficient intensity to penetrate deep tissues, including bone, and is therefore available for external detection.^11^ However, non-linear attenuation of photons resulted from tissue depth and tissue optical heterogeneity hampers the quantification of signal. Tissue scattering and absorption further limits the spatial resolution of bioluminescence 2D imaging.^11^ Another limitation is metabolic changes may influence the consistency of bioluminescence signal because luciferases often rely on ATP and cofactors. For example, it has been reported that in vivo bioluminescence signal may decline and even disappear even though the target cells still survive and express luciferase.^29^ Due to the above limitations, in bone tissue engineering studies, BLI is often used in association with another reporter, like fluorescent proteins, either to boost signal intensity or to double confirm BLI specificity.^12–15^ By adding fluorescent reporters, ex vivo flow cytometry or immunostaining analysis also becomes available, which may provide more insights for addressing the scientific question.

To overcome these challenges, BRET seems to be an attractive strategy because of enhanced signal intensity. BRET is a transfer of energy between a luminescence donor and a fluorescence acceptor under certain requirements.^30^ In our previous study, BRET-based GpNLuc reporter was created by an optimized fusion of the eGFP and NanoLuc moieties which enables efficient BRET (BRET ratio 2.60 ± 0.02).^16^ GpNLuc generates the brightest bioluminescent signal known to date: NanoLuc itself is >150-fold brighter than firefly and *Renilla* luciferases, and that GpNLuc has a near 10-fold increase in total light output over NanoLuc alone. GpNLuc significantly reduced image acquisition times and demonstrated exquisitely sensitive monitoring of tumorigenesis at deep tissue level. Following the successful application in cancer biology, now we are delighted to see the BRET-based GpNLuc also demonstrated impressive potential in bone tissue engineering, which widened the field of BRET applications. We have also created another LumiFluor reporter—LSSmOrange-NanoLuc (OgNLuc), which emits light at a longer wavelength (572 nm) that benefits tissue penetration, but generates weaker light than GpNLuc due to lower BRET efficiency.^16^ Thus, the GpNLuc reporter seems more suitable for a shallow subcutaneous location, for example, the calvaria defect described herein, whereas the OgNLuc may be more suitable for deep within the body.

Another potential advantage of using GpNLuc for BLI in bone tissue engineering is its independency on ATP. Inorganic phosphate homeostasis is crucial for hydroxyapatite formation and bone cell activities in the process of matrix mineralization. In vitro studies have demonstrated ATP has an impact on the differentiation and mineralization of osteoblasts.^31,32^ In addition, ATP level in cortical bone has been proposed as an indicator of bone healing, because of its correlation with the histological setting of bone repair.^18^ Therefore, using ATP-independent GpNLuc reporter may have minimized disturbance on the mineralization during bone defect repair and is more likely to acquire consistent bioluminescence data. In our study, it took no longer than 7 days for the signal from GpNLuc-rMSCs to generally reach stabilization (Figure 6(b)), which is much faster than the 30 days reported in Dégano et al.^12^ study that used ATP-dependent firefly luciferase. It is possible that the ATP-independency plays a role in this faster reachable stabilization, which may be valuable to bear in mind for future investigations.

In conclusion, by introducing BRET, this study offered an improved, robust, inexpensive bioluminescent cell tracking strategy for bone tissue engineering. Regarding future improvements, there are some valuable points worth noting. We found that crushed ex vivo samples generated much stronger bioluminescent signal than non-crushed samples (Figure 7(c) and (d)). Since crushing the sample shouldn’t release the cells from the scaffold into the media, the cell-generated BLI signal is still subject to potential effects of NFB absorption. Thus, the increase in signal is mostly likely coming from better luciferase substrate accessibility, which may be a critical factor to be considered for future improvement. During the bone regeneration process, continued extracellular matrix formation and mineral deposition forms a dense barrier that may prevent the diffusion of luciferase substrate. A combination of subcutaneous and intravenous injection may help to achieve thorough substrate infusion of the defect/repair site. Since bone defect also involves the destruction of blood vessels that further obstructs substrate delivery, the addition of angiogenic factors that benefits the vascularization in NFB may also help. At last, if detecting bioluminescence signal from deep within the body, it would be better to consider using our BRET-based OgNLuc reporter or novel furimazine analogs that provide red-shifted bioluminescence with NanoLuc to enable better signal penetration.^33^

## Supplemental Material

sj-docx-1-tej-10.1177_2041731421995465 – Supplemental material for Application of bioluminescence resonance energy transfer-based cell tracking approach in bone tissue engineering Click here for additional data file. Supplemental material, sj-docx-1-tej-10.1177_2041731421995465 for Application of bioluminescence resonance energy transfer-based cell tracking approach in bone tissue engineering by Lufei Wang, Dong Joon Lee, Han Han, Lixing Zhao, Hiroshi Tsukamoto, Yong-IL Kim, Adele M Musicant, Kshitij Parag-Sharma, Xiangxiang Hu, Henry C Tseng, Jen-Tsan Chi, Zhengyan Wang, Antonio L Amelio and Ching-Chang Ko in Journal of Tissue Engineering
