# Supplementary material for: Application of bioluminescence resonance energy transfer-based cell tracking approach in bone tissue engineering
Source: J Tissue Eng. 2021 Feb 16;12:2041731421995465. doi: 10.1177/2041731421995465 (PMC7894599; doi:10.1177/2041731421995465)
Supplement: sj-docx-1-tej-10.1177_2041731421995465 – Supplemental material for Application of bioluminescence resonance energy transfer-based cell tracking approach in bone tissue engineering [file sj-docx-1-tej-10.1177_2041731421995465.docx]

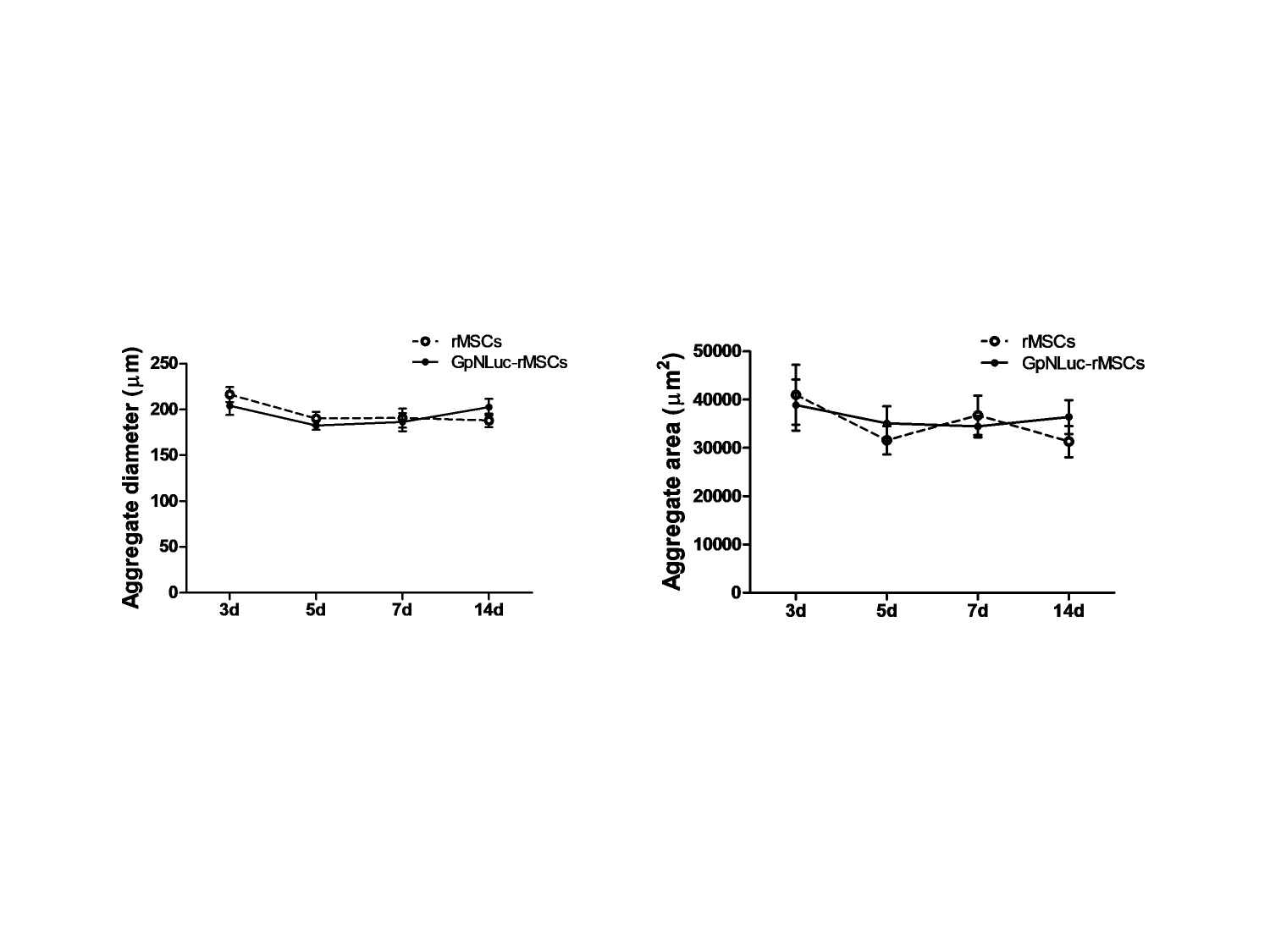


**Supplementary Fig. 1 Diameter and area measurement of cell aggregates.** Cell aggregates were growing in osteogenic medium for continual osteogenic induction. At various time points of osteogenic induction, the diameter and area of the aggregates was quantified using ImageJ. n = 6. Data shown as mean ± SEM.


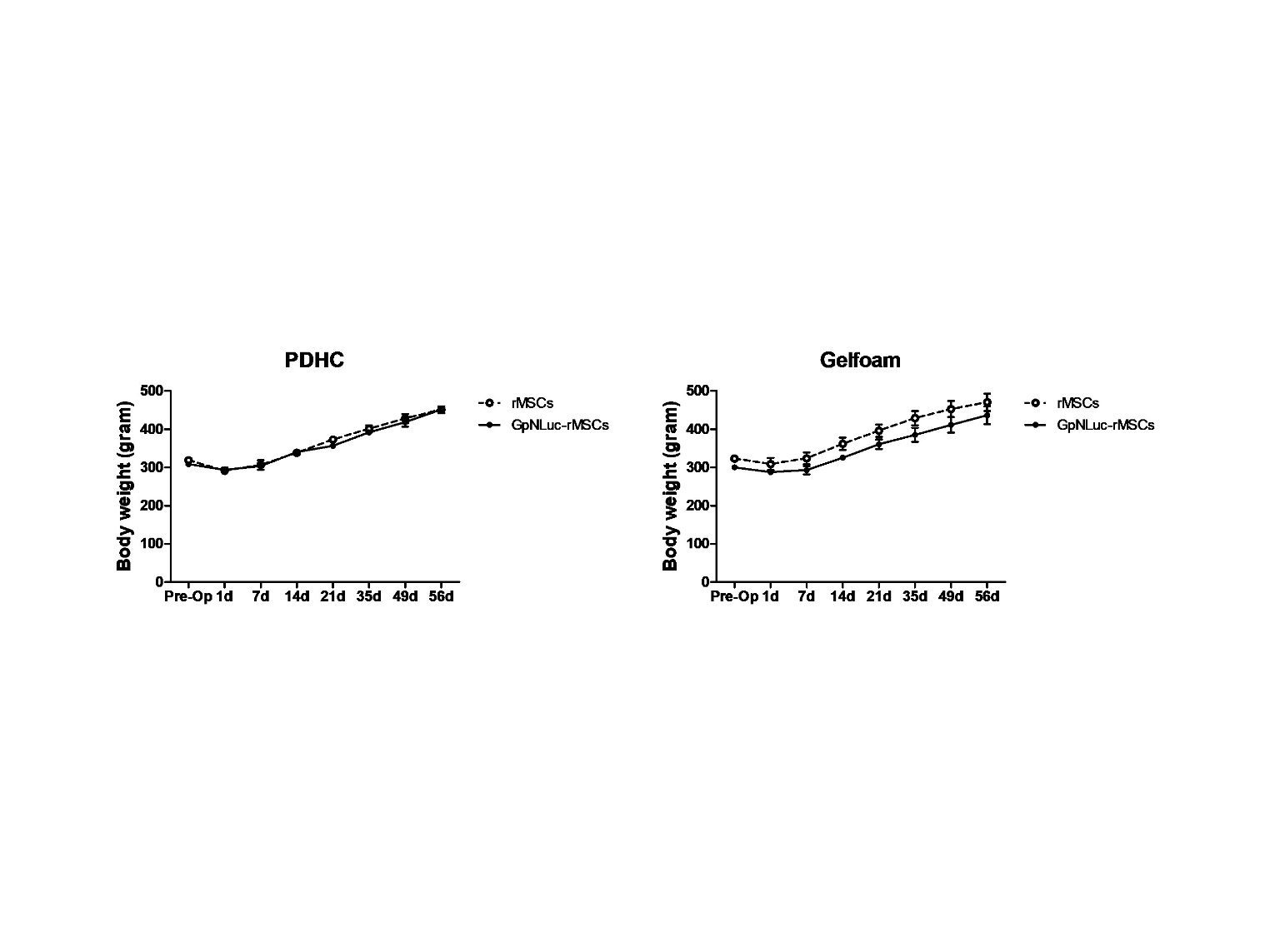


**Supplementary Fig. 2 Animal body weight.** Animal body weight was routinely recorded before and after the implantation of cell-scaffold constructs. n = 3. Data shown as mean ± SEM.

**Supplementary Table 1. Primer sequence for RT-qPCR**

| **Gene** | **Primer Sequence (5’-3’)** |
| --- | --- |
| *Bsp* | Forward: CCGGCCACGTACTTTCTT  Reverse: TGCACTGGAAACCGTTTCAGA |
| *Opn* | Forward: CCCATCTCAGAAGCAGAATCTT  Reverse: GTCATGGCTTTCATTGGAGTT |
| *β-actin* | Forward: GCAGATGTGGATCAGCAAGC  Reverse: CGACGCAAAATGTGGGAAAGA |
